# Oral Care Program for Successful Long-Term Full Mouth Habilitation of Patients with Hypohidrotic Ectodermal Dysplasia

**DOI:** 10.1155/2018/4736495

**Published:** 2018-11-29

**Authors:** Yuan-Lynn Hsieh, Michael Razzoog, Sabrina Garcia Hammaker

**Affiliations:** ^1^Resident, Graduate Prosthodontics, Department of Biological and Material Sciences, School of Dentistry, University of Michigan, Ann Arbor, Michigan, USA; ^2^Professor, Department of Biological and Material Sciences, School of Dentistry, University of Michigan, Ann Arbor, Michigan, USA; ^3^Clinical Assistant Professor, Department of Biological and Material Sciences, School of Dentistry, University of Michigan, Ann Arbor, Michigan, USA

## Abstract

Hypohidrotic ectodermal dysplasia (HED) is a rare congenital disorder that associates with dental manifestations of anodontia, hypodontia, and atrophic alveolar ridges. Although the disorder does not affect the life expectancy of the patient, it poses tremendous challenges on the patient's physical and psychosocial development. Early and multidisciplinary dental care can benefit HED children's development and improve their quality of life. This study presents two cases addressing the dental management continuously for 10 to 13 years in the dental school clinics. The keys to long-term success of the oral care program for HED patients at different age phases are reviewed and discussed, which can be summarized as early intervention, multidisciplinary collaborative care, and continuous recall.

## 1. Introduction

Ectodermal dysplasia (ED) refers to the developmental defects in the ectodermal-derived tissues such as the skin, hair, teeth, sweat glands, and thyroid gland [1]. The hypohidrotic ectodermal dysplasia (HED) is the most frequent subtype of ED and its incidence rate is estimated 1–7 per 100,000 live births [2]. A 15-year survey in Denmark reveals that the prevalence of HED is 21.9 per 100,000 people [3]. Three significant signs are commonly found in HED patients, including sparse hair, missing teeth, and abnormal sweat glands, which are also called “the clinical triad of HED” [4, 5]. Dental abnormalities, found in 79% of patients [2], is one of the primary clinical symptoms and signs of HED. Small-sized, cone-shaped teeth are commonly found in the maxillary central incisors and canines if the teeth are present [6, 7]. Moreover, the incidence of hypodontia and anodontia is reported higher in the mandibular dentition than that in the maxillary dentition [8].

The aforementioned dental defects associated with HED patients are largely not life threatening [9]. Nonetheless, dental professionals encounter a variety of challenges during the treatment course at different ages (Table 1). Firstly, congenital hypodontia or anodontia of the HED patients results in the urgent need of extensive oral habilitation at a young age. Secondly, any implemented dental therapy may require continuing adjustment according to children's growth [10]. Psychosocially, some HED children may experience a “nine-year crisis” which implies that they are more likely to be aware of the difference from their healthy peers and to have emotional and behavior disturbance [10]. Owing to early onset of extensive defects, dynamic change of oral condition, and psychosocial considerations, HED patients should have the access to a team of multidisciplinary specialists for persisting planning and treatment. This is one of the most important factors of long-term success [2, 11–13] spanning over from patients' young ages to adulthood. Facing challenges and difficulties in the oral care of HED patients, dental professionals need to know the proper oral care modalities at different age phases. To the best of our knowledge, most literatures related to the dental care of HED patients have focused on the individual treatment course and modifications. Herein, this report aims to present two HED patients under comprehensive oral care for 10 to 13 years with the introduction of an appropriate oral care program for the successful long-term treatment.

## 2. Case Report

**Figure 1:**
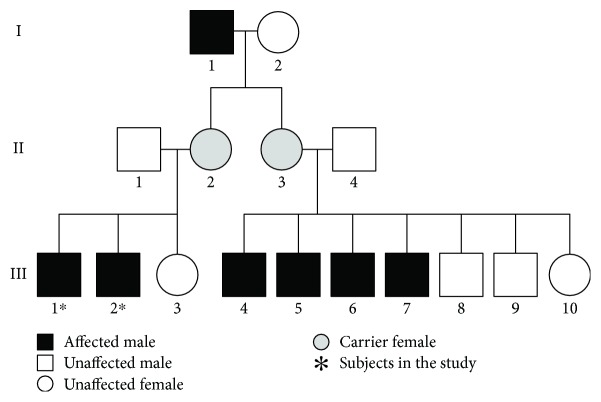
Pedigrees of HED in the families and relatives of the cases.

The HED is a rare congenital disorder inherited as an X-linked recessive trait and affecting more males than females [3, 6]. The pedigree investigation (Figure 1) of our patients is in line with this genetic feature. All the affected patients in generation III are male, and it could be tracked back to their grandfather (generation I-1) who also had the symptoms and signs of HED.

### 2.1. Subject 1

**Figure 2:**
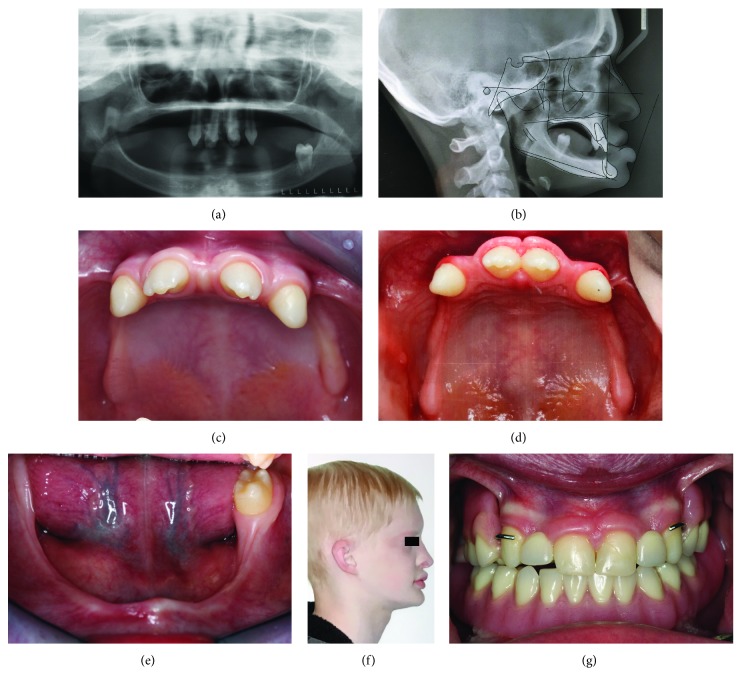
Clinic pictures of HED subject 1. (a) Panoramic radiograph at age twelve presented only five permanent teeth being developed on the patient. (b) Cephalometric radiography showed midface underdevelopment. (c) Patient presented with spacing teeth in the maxillary dentition before orthodontic treatment. (d) After orthodontic therapy (age fourteen), space between central incisors were closed. (e) Mandibular oligodontia was presented. Only left first molar was erupted. (f) Loss of vertical dimension of occlusion in profile view. (g) Front view of interim prosthesis in position.

The current 15-year-old Caucasian male patient diagnosed with HED and partial anodontia began his first dental visit at age five and received multidisciplinary dental care for the next ten years. At age five, he was evaluated by the pediatrician and referred to the Pedodontic clinic at the University of Michigan for oral habilitation. The psychological condition was within normal limit. Radiographs showed multiple congenitally missing teeth, including primary dentition and permanent dentition. Oral habilitation with interim partial denture was completed at age five. However, during the 3-year periodical recall, the first prosthesis became unfitted due to the rapid growth of the jaw bone. At the age of eight, another set of interim removable partial dental prostheses was fabricated at the Pedodontic clinic. When the patient turned 12, only five permanent teeth had erupted (Figure 2(a)) and retrognathic maxilla can be noted in the cephalometric radiograph (Figure 2(b)). He received orthodontic therapy which reduced midline diastema and provided more space to accommodate the replacement for the missing lateral incisors (Figures 2(c) and 2(d)). During the same period, gingivectomy and frenectomy were performed at the Periodontal clinic for enhancing orthodontic therapy. After finishing orthodontic treatment, he continued the oral habilitation at the Prosthodontic clinic. At age fourteen, he had only one molar in the mandible (Figure 2(e)) and did not have any occlusion pair of the teeth. Noticeably, the vertical dimension of occlusion (VDO) was lost (Figure 2(f)). Composite reconstruction was completed in both maxillary central incisors and a maxillary interim removable partial denture and a mandibular removable complete overdenture were fabricated in the Prosthodontic clinic (Figure 2(g)). Although the treatment of interim prosthesis was completed when the patient was 15 years old, further evaluation will be required in the future to consider a permanent prosthesis.

### 2.2. Subject 2

**Figure 3:**
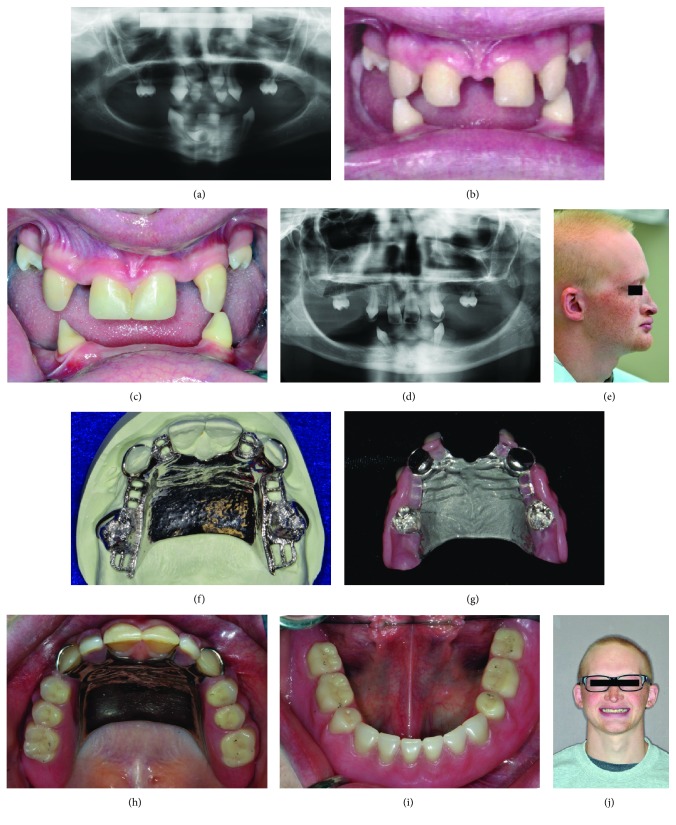
Clinic pictures of HED subject 2. (a) Panoramic radiograph at age seven showed tooth germ of permanent central incisors. All other permanent teeth were congenitally missing. (b) Frontal view at age twelve before orthodontic therapy. (c) Front view after completion of orthodontic and restorative treatment with composite veneers on maxillary canines and central incisors. (d) Panoramic radiograph at age eighteen. Underdeveloped alveolar ridge was noted in the edentulous area. (e) Loss of vertical dimension of occlusion and sparse hair and eyebrows are presented in the profile view. (f) The design of overlay cobalt-chrome alloy framework of maxillary prosthesis. (g) The tissue surface of the maxillary prosthesis. (h) Occlusal view of maxillary permanent removable partial denture in position. (i) Occlusal view of mandibular complete overdenture in position. (j) Finish of full mouth habilitation.

This 18-year-old Caucasian male patient, and who is the sibling of subject 1, was diagnosed with oligodontia, congenital hypothyroidism, and ectodermal dysplasia with missing sweat glands during childhood. At age five, he came to the University's pedodontic clinic for evaluation. His general health and psychological condition were within normal limit. However, because of abnormal sweat gland, his body cannot sweat and he has the problem of heat intolerance in high temperature environment or after severe exercise. Therefore, room temperature at the clinic was monitored in his visit. Beginning at age five, he had his first maxillary interim removable partial dental prosthesis fabricated at the Pedodontic clinic. As he grew up, the primary maxillary central incisors were the only shed teeth, and the rest of the primary teeth were preserved without succeeding permanent teeth (Figure 3(a)). The first prosthesis was replaced 5 years after fabrication. In order to accommodate the patient's new requirements, orthodontic therapy was suggested at age 13. Reduction of midline diastema was accomplished and composite veneers were done on the maxillary anterior teeth (Figures 3(b) and 3(c)). A maxillary Essix retainer was fabricated for replacement of lateral incisors. The appliance provided a satisfactory esthetic appearance during his early teenage years. Nevertheless, the patient was still lacking adequate chewing function due to missing posterior occlusion. His latest oral examination revealed only two permanent teeth and six deciduous teeth (Figure 3(d)). The vertical dimension of occlusion was diminished as well (Figure 3(e)). At age 18, a more definitive oral rehabilitation plan including a maxillary removable partial denture (RPD) and a mandibular overdenture was executed at the Prosthodontics clinic. Leveling of the posterior plane of occlusion was achieved with the fabrication of the maxillary RPD with an overlay cobalt-chrome alloy framework (Figures 3(f)–3(h)). For the mandibular overdenture prosthesis, both mandibular canines served as abutment teeth (Figure 3(i)). The vertical dimension of occlusion was increased by 5 mm to correct protuberant lips and gain restorative space. Patient was able to accommodate to the new prostheses in a short period of time and reportedly gained not only masticatory and esthetic functions (Figure 3(j)) but also confidence in his daily routine social relations.

## 3. Discussion

The dental therapy of HED patients is a long-term and active process which must be adapted to the growth of jaw bones [1]. It is widely recognized that the three most essential elements of oral habilitation for them are early intervention, multidisciplinary collaborative care, and continuous recall [2, 5, 6, 11, 14]. Along with different treatment modalities, many successful and satisfactory results had been reported in the literature [1, 5, 7, 11, 14, 15]. Since the oral care for HED patients is life-long, the challenges and considerations of oral habilitation vary at different stages of life, various oral care programs should be advised by the clinicians, and significant benefits are likely achieved. Herein, we present a review of appropriate oral care program for the successful long-term treatment of HED patients at different age phases (Table 1). During the preschool phase (under age 5), it is crucial to introduce a multidisciplinary dental team for early planning and intervention. With the aid of dental prosthesis improvements in nutrient intake, speech development, and appearance are widely reported [7, 14]. Although it may be difficult to execute dental treatment due to lack of cooperation at a young age, parents should be informed of the options and benefits of early dental care. Of note, the removable interim prosthesis is frequently used in this phase and has been named the “learning prosthesis” because it not only aids the HED child shift primary deglutition to normal mastication and swallowing but also helps them learn to deal with the oral challenge [5]. The multidisciplinary team of pedodontists and prosthodontists are the major specialists who usually administer the oral care program to the HED child at this stage [15]. During the primary school years (age 6–12), the deciduous teeth may shed while the maxilla and mandible bones continue growing and developing; therefore, periodic recall and adjustment of the first prosthesis are necessary for sustaining its usage [14]. The aim in this phase is to maintain the function and comfort of the dental prosthesis. Oral habilitation with interim prosthesis improves the appearance and may diminish the impact of social withdrawal effected by the “nine-year crisis” [10]. As the HED patient enters the adolescence phase (age 13–18), it is critical that the clinician considers not only the function and comfort of the dental prosthesis but also its esthetic components, since self-confidence in adolescence is more likely to be affected by facial appearance [16]. Most importantly, in the transition to permanent oral habilitation in adulthood, the adolescence phase is the ideal period to prepare the oral environment for future restoration. Adjuvant orthodontic therapy, which is advised to implement in this phase, assists in aligning the teeth and correcting the occlusion of the remaining dentition, thus setting the foundation for future permanent oral habilitation [2, 7, 17]. In the adult phase (age 19 and beyond), consideration of permanent oral habilitation such as the complete denture, removable partial denture, and implant-supported removable/fixed prostheses becomes practical for HED patients. It is important to mention that if implant therapy is considered, bone augmentation prior implant placement may be required due to the commonly present knife-edged alveolar ridges in HED patients [7, 18]. On the other hand, Le Fort I maxillary osteotomy may be executed to correct the midface deficiency and jaw discrepancy along with the ridge augmentation procedure [19, 20]. Notably, the implant success rate of HED patients, which attained 97.9%, does not significantly differ from the implants in the nonaffected population [18].

In summary, dental professionals encounter a variety of challenges during the treatment course of HED patients at different ages because of the dynamic change in the oral environment, as well as physical and psychological impacts which may be generated by the dental defects. To attain successful long-term full mouth habilitation, the implement of a comprehensive oral care program is advised at different age phases, including preschool, childhood, adolescence, and adulthood. Early dental intervention benefits the patients' physical and psychosocial development by improving their chewing, speech, and appearance. Maintaining the function, comfort, and aesthetics of the prosthesis is the steadfast objective of oral care from childhood to adolescence, requiring periodically recall. Every dental practitioner could play an important role in offering adequate oral care to affected children at a young age through a variety of dental modalities.

## Figures and Tables

**Table 1:** The challenges and advised oral care program for long-term success treatment of HED patient.

Phase	Age range and dental stage	Challenges and considerations in oral habilitation	Advised oral care program	Multidisciplinary dental specialists
(1) Preschool phase	(i) Younger than 5 (ii) Primary dentition	(i) Early diagnosis (ii) Child's cooperation in dental therapy (iii) Limited dental treatment options (iv) Child's hand dexterity to handle removable prosthesis (v) Maxillary prosthesis is in high priority in terms of appearance and verbal development (vi) Due to extensive defects and continuing growth, HED patient should have access to a team of multidisciplinary specialists for planning and treatment	**Goal: early intervention and accessibility to an interdisciplinary team of dental specialists** (i) Clinical, imaging, and genetic examination for early diagnosis (ii) Enhance child's familiarity with the environment of the dental clinic and inform the parents about short/long-term treatment options (iii) Fabrication of first interim denture prosthesis (“learning prosthesis”) (iv) Early oral treatment benefits HED child in better chewing function, adequate nutrient, normal appearance, and speech development	(i) Pedodontist (ii) Prosthodontist

(2) Childhood phase	(i) Age 6–12 (ii) Mixed dentition	(i) Affected patients begin to deal with distinct appearance among peers (ii) “Nine-year crisis” may cause social withdrawal (iii) Jaw bone growth and tooth shedding may cause the prosthesis to be gradually unfitted	**Goal: to maintain the function and comfort of first prosthesis** (i) Periodically recall to adjust existing prosthesis (ii) Replacement of existing prosthesis when necessary (iii) Implants on mandibular anterior area may be considered in fully edentulous patients	(i) Pedodontist (ii) Prosthodontist (iii) Periodontist (iv) Oral surgeon

(3) Adolescence phase	(i) Age 13–18 (ii) Early permanent dentition	(i) Esthetic considerations to strengthen self-confidence (ii) Interproximal space and malocclusion cannot be corrected solely by dental prosthesis (iii) Oral hygiene maintenance is difficult if orthodontic therapy is carried out	**Goal: preparation for permanent oral habilitation** (i) Periodically recall to adjust existing prosthesis (ii) Reshaping of cone-shaped teeth with direct composite restoration to enhance esthetics (ii) Orthodontic therapy to align residual teeth and correct occlusion (iv) Preservation of existing teeth and alveolar bone (v) Enhancement of oral hygiene and maintenance	(i) Pedodontist (ii) Orthodontist (iii) Prosthodontist

(4) Adult phase	(i) Age 19 and beyond (ii) Permanent dentition	(i) Extensive missing teeth and knife-edged alveolar ridge pose challenge in support, retention, and stability of permanent prosthesis, such as RPD or implants (ii) Skeletal growth is matured and favorable for permanent oral habilitation	**Goal: implementation of oral habilitation with permanent prosthesis** (i) Fabrication of permanent prosthesis with different modalities (complete denture, RPD, implant-supported removable/fixed prosthesis) (ii) Ridge augmentation may be necessary prior implant therapy	(i) Prosthodontist (ii) Periodontist (iii) Oral surgeon
